# The Psychiatric Case Register Middle Netherlands

**DOI:** 10.1186/1471-244X-11-106

**Published:** 2011-06-29

**Authors:** Hugo M Smeets, Wijnand Laan, Iris M Engelhard, Marco PM Boks, Mirjam I Geerlings, Niek J de Wit

**Affiliations:** 1 Julius Center for Health Sciences and Primary Care, University Medical Center Utrecht, Utrecht, The Netherlands; 2 Clinical and Health Psychology, Utrecht University, Utrecht, The Netherlands; 3 Altrecht, Institute for Mental Health Care, Utrecht, The Netherlands

## Abstract

**Background:**

The Psychiatric Case Register Middle Netherlands (PCR-MN) registers the mental healthcare consumption of over Dutch 760,000 inhabitants in the centre of the Netherlands. In 2010 the follow-up period was over ten years. In this paper we describe the content, aims and research potential of this case register.

**Description:**

All mental healthcare institutions in the middle-western part of the province of Utrecht participate in the PCR-MN case register. All in- and out-patients treated in these institutions have been included in the database from the period 2000 to 2010. Diagnosis according to DSM-IV on axis I to IV, visits to in- and out-patient clinics and basic demographics are recorded. A major advantage of this register is the possibility to link patients anonymously from the PCR-MN cohort to other databases to analyze relationships with determinants and outcomes, such as somatic healthcare consumption, mortality, and demographics, which further increases the research potential

**Conclusions:**

The PCR-MN database has a large potential for scientific research because of its size, duration of follow-up and ability to link with additional databases, and is accessible for academic researchers.

## Background

Psychiatric case registers (PCRs) are described as 'patient-centered longitudinal records of contacts with a defined set of psychiatric services originating from a defined population'[1]. In Denmark systematic collection of psychiatric data existed as early as 1938 and computerized recording of data started in 1969 [2]. The first registers in Great Britain arose around the 1960s. The Netherlands originally had three PCRs, one in the north, one in the south, and one in the west, of which the oldest goes back to the 1970s [3]. Around the end of the 1990s a fourth PCR was founded; the Psychiatric Case Register Middle Netherlands (PCR-MN). From the beginning this PCR has focused on the potential linkage of its registered mental health data to additional sources of data. This paper aims to describe the PCR-MN case register and its potentials for research to inform researchers and invite them to cooperate in scientific research.

### Purpose of the Psychiatric Case Register

Traditionally, mental healthcare in the Netherlands was funded by a special social legislation programme, separate from the general medical care funding. Given the open-end, lump sum based funding, mental health institutions used to record psychiatric patient management inadequately. Because of the need for a better understanding of the psychiatric patient population, the need for analysis of treatment efficacy and increasing costs, regional databases on patients treated in mental health care were founded. The Dutch government provided financial support for the development of such databases, because they provided detailed information about the morbidity, characteristics and costs of mental health provision. In the last decade the demand for good healthcare information has been reinforced by changes of the funding system into individual reimbursed expenses. Therefore the mental healthcare institutions in the central province of Utrecht created the fourth case register, with data from 1999 onwards, offering opportunities for epidemiologic research in psychiatry. To strengthen the infrastructure and facilitate research within the database the case register was incorporated in the Julius Center for Health Sciences and Primary Care of the University Medical Center Utrecht in 2008 and now receives a structural funding from the Dutch Ministry of Health.

## Construction and Content

### The study area

The PCR-MN database covers the mid-western part of the province of Utrecht, the Netherlands and included over 760,000 inhabitants in 2010. This is a representative sample of 5% of the total Dutch population in urban, semi-rural and rural areas, including the main city of Utrecht. Approximately 10% of the population is of non-Dutch origin. From 2011 the database will be extended with mental health institutions in the north-eastern part and will cover then the whole province of Utrecht with over 1.2 million inhabitants

### The participating centers

All mental health care institutions in the region participate in the PCR-MN. All patients who consult these institutions and are treated are included in the database, both in and outpatients. The psychiatric hospitals, Altrecht Mental Health Care, St. Antonius Hospital and University Medical Center, focus on all psychiatric disorders, both for patients with and without a referral from the general practitioner and provide both inpatient and outpatient care. Sixty private psychotherapists are also affiliated with PCR. Center Maliebaan is the main provider of addiction services and two non-profit foundations, Kwintes and SBWU, provide the assisted living in the PCR-MN region. The GG&GD Utrecht (municipal health authority) monitors the mental and social healthcare of homeless, socially isolated or other maladjusted people in the in the city of Utrecht. Over the last years about 60,000 patients were registered annually in the database of which 75% was treated in Altrecht MHC. Furthermore the database contains about 5000 addicted patients and 6000 persons in assisted living situations.

Representatives of the participating institutions serve on the advisory board of the PCR-MN in which they advise on the policy of the register and the use of data for research.

### Data recorded in the PCR-MN

At the beginning of each calendar year, all information on demographic characteristics, diagnosis and type of care provided is collected from the participating centers. Basic demographics include date of birth, gender, the first part of the postal-code, country of birth of the patients and country of birth of his or her parents. Both the diagnosis at intake and at discharge are recorded using the DSM-IV axis I to IV[4]. Table 1 presents the number of patients per year within the main DSM-IV axis I and II chapters, for citizens of the area covered by the PCR-MN. As can be seen here, the most frequently diagnosed disorders are mood disorders and overall, the number of patients per diagnostic chapter is increasing over the years. The type of care is recorded per day on an individual level, and it is possible to follow individual patients over the participating hospitals (Table 2).

**Table 1:** Total cases with the most common DSM-IV axis one and two diagnoses.

	Chapter	2002	2003	2004	2005	2006	2007	2008	2009
**Number**	**Name**	**PCR**	**PCR**	**PCR**	**PCR**	**PCR**	**PCR**	**PCR**	**PCR**

1	Disorders usually first diagnosed in childhood	1,010	1,305	1,661	2,074	2,555	2,730	4,490	4,262

2	Delirium, dementia, and amnestic and other cognitive dis.	519	644	913	1,243	1,663	1,675	1,891	1,925

3	Mental disorders due to a general medical condition NEC	43	68	70	82	155	114	177	209

4	Substance-related disorders	1,453	1,093	1,612	3,179	3,438	2,804	5,691	4,764

5	Schizophrenia and other psychotic disorders	969	1,097	1,270	1,624	2,588	1,677	1,900	1,758

6	Mood disorders	3,519	4,198	4,913	5,504	6,581	6,271	7,189	6,586

7	Anxiety disorders	1,676	2,135	2,620	3,043	3,520	3,593	4,269	4,359

8	Somatoform disorders	538	651	818	974	974	1,163	1,296	1,307

9	Factitious disorders	6	8	10	12	16	11	20	20

10	Dissociative disorders	64	65	103	108	141	145	147	144

11	Sexual and gender identity disorders	530	711	907	1,036	953	1,445	1,943	1,952

12	Eating disorders	365	403	562	772	845	842	988	1,061

13	Sleep disorders	29	28	47	63	44	96	122	159

14	Impulse-control disorders NEC	174	190	246	348	355	444	640	640

15	Adjustment disorders	904	1,250	1,567	1,756	1,896	2,711	3,639	4,070

16	Personality disorders (Axis II)	1,543	1,820	2,293	2,815	3,648	3,311	4,980	5,051

A diagnosis was counted when it was made during the specified calendar year or the year prior to the specified calendar year.

**Table 2:** Total patients receiving inpatient, outpatient, crisis or assisted living care per year for the main categories of care

	2002	2003	2004	2005	2006	2007	2008	2009
Inpatient care	3,693	3,696	4,245	4,547	4,691	5,304	5,673	5,629

Outpatient care	24,236	26,092	32,467	34,738	37,768	44,809	49,012	51,914

Crisis care	3,379	3,612	4,069	4,204	3,936	4,133	4,144	4,691

Assisted living	891	1,123	1,769	2,056	2,510	4,557	5,328	5,436

### Ethics and privacy

Dutch privacy law allows the use of personal (healthcare) data on behalf of scientific research if a number of demands are met. The most important one is that individual patient data can not be traced to an individual. In the PCR-MN database, identifying variables are encrypted, which makes it impossible for researchers to identify individual patients. A research review commission decides whether a request of an investigative body for the delivery of data for research can be met. Datasets are made available strictly under the condition of anonymity, safe data storage and inaccessibility to others. After use, the destruction of all data sets is mandatory.

### Additional data for research

The large size of PCR-MN data only on diagnoses and treatment are insufficient for most observational research projects. Studies such as intervention and prognostic studies can be performed in cohort and case-control designs, but need more determinants and confounding variables in their analyses. Potential linkage to other sources of data can be used to enrich the database, which can supplement the available clinical information and patient characteristics. The most important databases for this data linkage are the databases of Agis Health Insurance Company (Agis), which is the largest healthcare insurer in the province of Utrecht, Statistics Netherlands with information of all residences and the Network of General Practitioners within the Utrecht region.

#### Linking with Agis Health Database

Agis Health Insurance Company (Agis) is the main health insurance company in the central part of the Netherlands. In the Agis Health Database (AHD) the payments for the provision of all medical care to its insured patients is recorded. This includes extensive records of all outpatient diagnostic and therapeutic interventions, such as all drugs delivered by pharmacists, health services delivered by hospitals, general practitioners and other primary care professionals. The AHD is described extensively elsewhere [5].

**Figure 1:**
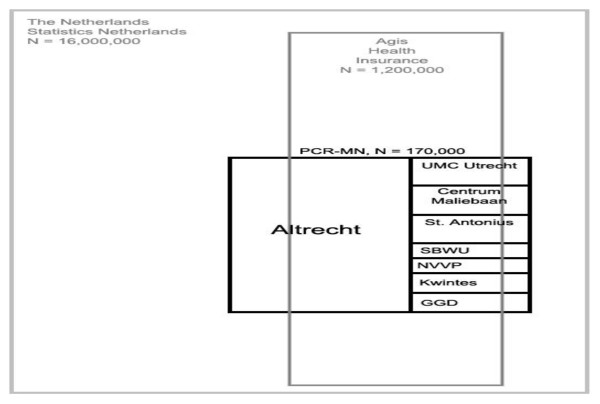
**Schematic overview of the overlap of the PCR-MN, Agis Health Database and Statistics Netherlands**.

Around two third of all PCR-MN patients are insured by Agis (Figure 1). Agis has a large proportion of clients from lower socioeconomic standard which may result in an overrepresentation in a group successfully linked patients. Once individual case linkage is established, all reimbursed healthcare intervention can be retrieved from the AGIS database. By linking the psychiatric healthcare from the PCR-MN to somatic healthcare consumption, it is, for example, possible to evaluate the influence of somatic co-morbidity on the effectiveness of psychiatric interventions.

#### Linking with Statistics Netherlands

In the Netherlands, Statistics Netherlands (CBS) is responsible for collecting and processing data in order to publish statistics to be used in practice, by policymakers and for scientific research. The published information of Statistics Netherlands incorporates a multitude of societal aspects, such as the incomes of individual people and their education [6]. Statistics Netherlands makes its datasets available for scientific research under the condition of strict confidence, and without any identifying variables. Linking of cases from the PCR-MN case register to the database of CBS leads to a match around 80% of all cases (Figure 1). A double match often occurs when two or more persons live in the same postal-code area. Analyzing data from Statistics Netherlands used to be done by a remote access terminal and data can never be downloaded (see their homepage, http://www.cbs.nl/en-GB).

#### Linking with General Practitioners Network

From 2011 several smaller General Practitioner Networks will join forces in the large Julius General Practitioner Network (JHN) that has stationed its database in the University Medical Center of Utrecht. The network contains over 300.000 residences in the Utrecht region, which makes it attractive for matching cases of the PCR-MN database. The JHN data contain the diagnostic codes (ICPC) of all consulting patients, prescribed drugs (ATC) as well as referral and laboratory information. In the near feature matching procedures will be elaborated, so linkage with the PCR-MN will become easy for study purposes.

## Utility and Discussion

### Studies using the Psychiatric Case Register Middle Netherlands

Routine databases like the PCR-MN are attractive for research because of their large size, longitudinal perspective, and practice-based information. Several studies have already been conducted using data from the PCR-MN case register. From an epidemiological perspective, etiological, intervention and prognostic studies are feasible with and without using links to other sources for additional information. Below we will give a number of examples using the linked PCR-MN database.

One study examined whether being diagnosed with both an anxiety disorder and a depression increases the hazard-ratio for death during follow-up compared to only one of these disorders. All cases were linked to the causes of death register of Statistics Netherlands in a 10 year follow-up cohort study, together with a random control group, after the initial diagnosis. An increased risk was demonstrated in patients with an anxiety disorder and in patients with a depression. However, the hazard ratio among people with both disorders was similar to those with only a depression (Laan W, Termorshuizen F, Smeets HM, Boks M, Wit NJ, Geerlings MI. A comorbid anxiety disorder does not result in an excess risk of death above the risk of death associated with a depressive disorder alone. *Submitted *). A second study was performed on the risk of several psychiatric disorders among minorities in Utrecht compared to Dutch natives. All cases with a relevant psychiatric diagnosis were subsequently linked to their birth records in the database of Statistics Netherlands. The findings in this cohort study showed significantly increased relative risks for treatment because of a depression or a non-affective psychosis among all immigrant groups [7].

In another case-control study, we investigated whether the use of corticosteroids was associated with a decreased risk of subsequent psychosis. All glucocorticosteroid prescriptions prior to the first registered psychotic episode in patients from the PCR-MN database were extracted from the AHD. Then the number of defined daily doses (DDDs [8]) was compared to a control group and the results showed that the risk of psychosis was lower in those who have used these drugs [9].

In a prognostic study at last the effect of non-compliance to anti-psychotic drugs on the risk of a psychotic relapse was measured. From all cases of the PCR-MN database that were discharged from an inpatient clinic of Altrecht, the reimbursed antipsychotic drugs were extracted from the AHD. The results of this study indicated a significantly increased risk for relapse with increasing non-compliance using a continuous compliance scale [10].

### Strengths and weaknesses

A major strength of the PCR-MN database is that it enables researchers to do longitudinally studies without interfering with regular care, thus allowing cohort studies nested within the PCR-MN main cohort, but also nested case-control studies. In a nested cohort, patients are selected based upon a determinant in the cohort; in a nested case-control study, patients are sampled based on the outcome.

Another strength is that the PCR-MN covers all psychiatric healthcare provided in the region. Patients referred to another psychiatric hospital in the region can still be identified in the case-register.

Also, because the registration of diagnosis and psychiatric contacts is based upon the financial databases of the psychiatric centers, the registration is very accurate. If the diagnoses are not registered well the costs will not be reimbursed by the insurance company.

Finally, because the PCR-MN case register is one of four Dutch psychiatric case-registers it is also possible to compare results from the Utrecht region to other regions covered by one of the other case-registers or even to analyze aggregated data from the four PCRs together.

A disadvantage of the registration in the PCR-MN is that some patient characteristics are subject to changes during follow-up. For example, the postal-code can result in loss to follow-up, because a patient may move to another part of the region and will not longer be treated at the same psychiatric hospital. The particular patient would then be identified as a new patient increasing the incidence and prevalence of disorders in the region. Also, patients who die or move from the region are not reported as such to the PCR-MN. It is difficult to determine to what extent this loss to follow-up occurs

A second limitation is that diagnoses in the PCR-MN are clinical diagnoses and not the result of a standardized structured diagnostic questionnaire. Also the reason to terminate psychiatric healthcare is not registered i.e. it is unknown if the patient is cured.

### Future developments

From 2010 onward we aim to also include the Axis 5 (Global Assessment of Functioning (GAF) score) of the DSM-IV in the PCR-MN database. The GAF score is a numeric scale with a range of 0 to 100 that aims to describe the social, occupational, and psychological functioning of a person.

In the coming years, the identification of patients in the database based upon the gender, postal code and date of birth will be replaced by a registration based upon the Dutch social security number (BSN). This requires the use of a 'third trusted party' to protect the privacy of patients in the case-register, which is currently being explored.

## Conclusions

The Psychiatric Case Register Middle Netherlands registers the psychiatric healthcare consumption and diagnosis of over 750,000 inhabitants. The PCR-MN now has a follow-up period of ten years. To aid scientific research, cases from this database can be linked to other databases covering roughly the same population. By linking these databases, psychiatric healthcare consumption, somatic healthcare consumption and important demographic variables can be studied. This resource is instrumental in psychiatric epidemiological studies and facilitates cohort and case-control studies.

## Availability and requirements

The PCR-MN database is available for academic research only. Scientists are encouraged to submit research proposals in which data from the PCR-MN can be used. To guarantee the feasibility and originality of the research performed and the privacy of those in the database all research proposals will be evaluated by a research review commission. The PCR-MN researches can be contacted through the corresponding author.

## List of abbreviations

AHD: Agis Health Database; ATC: Anatomical Therapeutic Chemical Classification System; CBS: Statistics Netherlands; DDD: Defined Daily Doses; DSM-IV: Diagnostic and Statistical Manual of Metal Disorders; GG&GD: Municipal Public Health Department; ICPC: International Classification of Primary Care; JHN: Julius General Practitioner Network; PCR-MN: Psychiatric Case Register Middle Netherlands; UMC: University Medical Centre

## Competing interests

The authors declare that they have no competing interests.

## Authors' contributions

HMS and WL designed the content structure and provided the figures of the description of the PCR-MN. IE and MB represent the psychiatry institutions and contributed to the content of the data. MG and NdeW represent study groups of the PCR-MN and contributed to the utility of the database. All authors read and approved the final manuscript.

## Pre-publication history

The pre-publication history for this paper can be accessed here:

http://www.biomedcentral.com/1471-244X/11/106/prepub
